# Traditional and Web-Based Technologies to Improve Partner Notification Following Syphilis Diagnosis Among Men Who Have Sex With Men in Lima, Peru: Pilot Randomized Controlled Trial

**DOI:** 10.2196/jmir.9821

**Published:** 2018-07-03

**Authors:** Jesse L Clark, Eddy R Segura, Catherine E Oldenburg, Hector J Salvatierra, Jessica Rios, Amaya Gabriela Perez-Brumer, Pedro Gonzales, Bhupendra Sheoran, Jorge Sanchez, Javier R Lama

**Affiliations:** ^1^ David Geffen School of Medicine Division of Infectious Diseases, Department of Medicine University of California, Los Angeles Los Angeles, CA United States; ^2^ Escuela de Medicina Universidad Peruana de Ciencias Aplicadas Lima Peru; ^3^ Francis I Proctor Foundation University of California, San Francisco San Francisco, CA United States; ^4^ Asociacion Civil Impacta Salud y Educación Lima Peru; ^5^ Mailman School of Public Health Department of Sociomedical Sciences Columbia University New York, NY United States; ^6^ YTH San Francisco, CA United States; ^7^ Centro de Investigaciones Tecnológicas, Biomédicas y Medioambientales Universidad Nacional Mayor San Marcos Lima Peru

**Keywords:** infectious diseases, syphilis, partner notification, men who have sex with men, Latin America

## Abstract

**Background:**

Patient-initiated partner notification (PN) following the diagnosis of a sexually transmitted infection is a critical component of disease control in men who have sex with men (MSM) sexual networks. Both printed and internet-based technologies offer potential tools to enhance traditional partner notification approaches among MSM in resource-limited settings.

**Objective:**

This randomized controlled trial aimed to evaluate the effect of 2 different PN technologies on notification outcomes following syphilis diagnosis among MSM in Peru: a Web-based notification system and patient-delivered partner referral cards.

**Methods:**

During 2012-2014, we screened 1625 MSM from Lima, Peru, for syphilis infection and enrolled 370 MSM with symptomatic primary or secondary syphilis (n=58) or asymptomatic latent syphilis diagnosed by serology (rapid plasma reagin, RPR, and Microhemagglutination assay for Treponema pallidum antibody; n=312). Prior to enrollment, potential participants used a computer-based self-interviewing system to enumerate their recent sexual partnerships and provide details of their 3 most recent partners. Eligible participants were randomly assigned to one of 4 intervention arms: (1) counseling and patient-initiated Web-based PN (n=95), (2) counseling with Web-based partner notification and partner referral cards (n=84), (3) counseling and partner referral cards (n=97), and (4) simple partner notification counseling (control; n=94). Self-reported partner notification was assessed after 14 days among 354 participants who returned for the follow-up assessment.

**Results:**

The median age of enrolled participants was 27 (interquartile range, IQR 23-34) years, with a median of 2 partners (IQR 1-5) reported in the past month. Compared with those who received only counseling (arm 4), MSM provided with access to Web-based partner notification (arms 1 and 2) or printed partner referral cards (arms 2 and 3) were more likely to have notified one or more of their sexual partners (odds ratio, OR, 2.18, 95% CI 1.30-3.66; *P*=.003 and OR 1.68, 95% CI 1.01-2.79; *P*=.045, respectively). The proportion of partners notified was also higher in both Web-based partner notification (241/421, 57.2%; *P*<.001) and referral card (240/467, 51.4%; *P*=.006) arms than in the control arm (82/232, 35.3%).

**Conclusions:**

Both new Web-based technologies and traditional printed materials support patient-directed notification and improve self-reported outcomes among MSM with syphilis. Additional research is needed to refine the use of these partner notification tools in specific partnership contexts.

**Trial Registration:**

ClinicalTrials.gov NCT01720641; https://clinicaltrials.gov/ct2/show/NCT01720641 (Archived by WebCite at http://www.webcitation.org/70A89rJL4)

## Introduction

Initially developed in the 1930s as a technique for syphilis control in the United States, notification of sexual partners following the diagnosis of a sexually transmitted infection (STI) remains central to efforts aimed at controlling the spread of HIV and other STIs in at-risk populations [1]. By retracing existing transmission pathways, partner notification (PN) offers the possibility to target efforts to the specific sexual networks structuring the spread of disease in a population [2,3]. Partner-based strategies also provide an opportunity to access individuals and sexual networks that remain unaccessed by traditional HIV or STI education and prevention interventions, such as men who have sex with men (MSM) but do not identify as gay or bisexual [4-6]. The recent resurgence in syphilis infection among MSM in the United States and Europe (and the persistently high prevalence of syphilis in the global South) highlights the importance of developing and refining partner-based efforts to identify and treat syphilis transmission networks in high-risk populations [7-9].

In the field of PN, 3 different types of notification are commonly recognized: (1) provider-initiated notification, in which professional counselors and newly diagnosed individuals work together to identify and notify recent partners; (2) patient-directed notification, in which the index case is encouraged to independently notify their partners after a brief counseling intervention; and (3) contract notification, in which the responsibility for notification shifts from patient to provider after a specified time period [10]. While provider notification is generally more effective than patient-directed efforts, resource-limited health systems in low- and middle-income countries (LMIC) often lack the personnel necessary to conduct detailed contact-tracing studies and so depend primarily on patient-based notification [11-14].

To characterize patient-initiated notification systems, Ferreira et al differentiated between simple and enhanced patient referral [10]. In the simple patient referral, PN is encouraged through professional counseling at the time of diagnosis that emphasizes the importance of notifying recent partners. In the enhanced referral, supplementary tools, including educational video- and theory-based counseling interventions, patient-delivered partner treatment, referral cards, or Web-based notification tools, could be used to motivate and support patient-directed notification efforts [15-18]. Although these standardized categories have formed the central paradigm for PN research, additional research is needed to address overlap and intersection between different methods of notification in real-life clinical populations.

Previously, printed partner referral cards have been used in various circumstances and appear to be effective in promoting notification within heterosexual partnerships [19,20]. However, evidence of the effect of referral cards in developing country settings is limited, and their use within MSM partnerships has not, to our knowledge, been evaluated in any previous randomized controlled trials (RCTs) [21-23].

Data the on the use of internet-based PN systems is mixed. Surveys of STI clinic patients in the United States and Australia, as well as MSM internet users in the United States, have found high levels of acceptability for Web-based notification systems [24-26]. In Europe and Australia, provider-managed internet notification has also been found to be useful in supporting PN during routine clinical practice [27-29]. In our recent study of STI-diagnosed MSM from Lima, Peru, the availability of a website that could deliver anonymous notification messages was expected to significantly increase the notification among men who were expected to not inform their partners under existing conditions [30]. In contrast, previous studies from the United States assessing public awareness and the actual use of PN websites among community-based samples of MSM, as well as male and female visitors to STI clinics, found low levels of penetration into these target populations [31,32]. The only RCT data on the effectiveness of Web-based PN among MSM comes from an incomplete study in Seattle (Washington, USA) that found poor uptake and low levels of acceptability among potential recipients of Web-based notification messages [33]. To the best of our knowledge, no other study has conducted head-to-head comparisons of Web-based systems with other PN technologies [34].

To address this gap in knowledge, we assessed the effect of new and traditional PN technologies on self-reported PN outcomes among MSM recently diagnosed with syphilis infection in Lima, Peru. While the Peruvian Ministry of Health maintains detailed guidelines for managing sexual partners of individuals with syphilis (including provider counseling, distribution of referral cards, and home treatment visits for partners of pregnant women), these services are delivered inconsistently in practice [35,36]. The deficiencies of existing partner management systems in Peru can be seen in the findings from a previous study where after 1 year, only 41% of individuals with syphilis infection had notified any of their partners and 43% had been reinfected after the confirmation of cure [37,38]. We conducted a factorial, RCT to compare the effect of printed partner referral cards and/or access to a Web-based notification system against standard counseling on self-reported PN outcomes among MSM in Lima, Peru, with untreated syphilis infection.

## Methods

### Study Design

Between November, 2012 and July, 2014, we conducted a four-arm, factorial RCT (NCT01720641) to assess the effect of new PN tools on notification outcomes among MSM in Lima, Peru, with untreated syphilis infection. The 4 arms included were (1) printed referral cards; (2) Web-based PN; (3) both printed referral cards and Web-based notification; and (4) control (standard of care) counseling procedures.

### Screening Procedures

Potential participants were recruited from both community and HIV or STI clinic sites by the staff of the Asociacion Civil Impacta Salud y Educacion (Lima, Peru). Both men and transgender women (TW) who reported anal intercourse with at least one male or transgender female partner in the previous 6 months were invited to participate in an STI screening protocol. Participants in the screening study completed a computer-assisted self-administered (CASI) survey that addressed demographic characteristics, history of HIV and STIs, alcohol and drug use, and sexual network characteristics, as well as attitudes, beliefs, and perceived community norms regarding PN for HIV and STIs. The survey also asked for detailed characteristics of participants’ 3 most recent sexual partners, including each partner’s gender and sexual identity, sexual practices performed with the partner during the last encounter, and the likelihood of notifying the partner in the event of an STI diagnosis. In order to assist with future recall of partner data, participants were asked to identify each of these 3 partners with a nickname or other identifying characteristic (eg, “the guy in the blue shirt from *La Cueva* ”).

All participants underwent a physical examination to identify signs of primary or secondary syphilis infection (painless ulcerative lesions on oral, anal, or genital mucosa or macular rash suggestive of secondary syphilis). Following the clinical examination, participants’ blood samples were collected to test for syphilis infection using the rapid plasma reagin (RPR) assay (RPRnosticon; Biomérieux) with microhemagglutination assay for Treponema pallidum antibodies (MHA-TP) confirmation (MHA-TP; Organon Teknika) and serial dilution of positive RPR titers. Although all participants were offered free HIV testing, it was not required as a condition of enrollment. The results of laboratory assays were provided within 2 weeks of screening.

Participants with syphilis infection were treated according to the stage of infection, as determined by a study physician following the review of participants’ history of syphilis infection, antibiotic treatment, and RPR titer(s). Cases of symptomatic primary, secondary, and early latent syphilis infection were treated with a single intramuscular injection of 4.2 million units penicillin G benzathine. Conversely, cases of asymptomatic late latent infection were treated with 3 weekly injections of 2.4 million IU penicillin G benzathine. However, participants with newly diagnosed HIV infection were referred to local HIV treatment centers for the initiation of free antiretroviral therapy provided by the Peruvian Ministry of Health.

### Randomization and Enrollment

We enrolled MSM and TW diagnosed with untreated syphilis (primary, secondary, or latent infection for which the treating physician recommended antibiotic therapy). Participants with symptomatic evidence of primary or secondary infection were enrolled at the initial screening visit, while participants with asymptomatic infection diagnosed by serology were enrolled after receiving the results of their RPR or MHA-TP testing. All participants provided signed informed consent for a study of, “If and how men with an STI inform their recent sexual partners of their diagnosis.” Next, we assigned participants in a 1:1:1:1 fashion to one of the 4 study arms according to a predefined 400-subject randomization scheme generated by the first author at the website (www.random.org).

### Intervention and Control Procedures

Each randomization envelope contained an assignment to one of the following 4 arms: (1) Web-based PN, (2) referral cards, (3) combined referral cards and Web-based PN, or (4) control. All allocation assignments were concealed in sealed, opaque, sequentially numbered envelopes that were opened in a numerical order by the study counselor at the point of randomization. To ensure visual and physical consistency of sealed randomization envelopes and to maintain concealment prior to allocation, each envelope was filled with a written study arm assignment, 1 PN counseling script, 5 partner referral cards or blank sheets of paper of the same color and consistency as the referral cards, and 1 Web-based PN access card or a blank note card of the same color and consistency. No deviation from the sequential allocation order or wasting of randomization envelopes was reported.

Randomization envelopes for all 4 arms included a standardized script that was read verbatim by the counselor. The counseling script advised the participants about the importance of notifying their recent partner of their STI diagnosis and informed them of the availability of free testing and treatment resources at the study website, as well as at other area health centers. Participants were also reminded that their safety was paramount and so they should not attempt to notify any partner who might react with violence or abuse.

Participants in the control arm did not receive any additional counseling or PN tools. Participants in the referral cards arm were provided with 5 printed cards to be delivered to a maximum of 5 of their recent sexual partners. Each card contained information about the symptoms and sequelae of syphilis infection, as well as the locations and operating hours of local sites offering free or low-cost HIV and STI testing services. Participants in the Web-based PN arm were read a brief script describing the Web-based notification resources available at (URL:http://www.inspot.org; Accessed: 2018-01-11) created by YTH, a nonprofit organization designed to use technology to promote youth health and wellness. A Spanish language “Peru” section of the inSPOT website was created specifically for this study and not publicized outside of the trial. Website content was not modified after initiation of the trial. In addition to providing anonymous PN messaging services, the Peru section provided information on testing and treatment resources available in major metropolitan areas of the country. Participants in the Web-based referral arms were provided with a note card indicating the website address. Participants in the combination referral cards/Web-based PN arm were provided with both printed referral cards and access to the inSPOT website using the methods described above.

### Endpoint Assessment

Participants in all four arms were asked to return to the clinic in 14-21 days for a follow-up evaluation. The prespecified primary endpoint was self-reported PN. At the follow-up visit, participants completed a brief CASI survey to assess how many of their recent partners (from the 30 days before screening) had been notified, as well as whether each of their 3 most recent partners had been notified and received antibiotic treatment. Participants were reminded of the total number of sexual partners that they had reported at the baseline visit and asked to quantify how many of these partners had been notified. To assist with recall of data for the 3 most recent partners, participants were reminded of the nickname or other identifying characteristic they had assigned each partner, as well as partners’ gender and sexual identity. Furthermore, survey questions asked whether each partner had been notified and used a 4-point Likert scale to assess the participants’ degree of certainty for whether the partner had received the notification message, whether the partner had sought HIV or STI testing, and whether the partner had received any STI treatment. Operational statistics on the use of the inSPOT.org website during the study period were collected by the YTH staff.

### Sample Size and Power Calculations

Sample size calculations were based on previous observational studies of Peruvian men and women diagnosed with HIV or STI [12,39]. Assuming a baseline frequency of 56% for notification of any partner, a sample of 100 subjects per arm was projected to have 80% power to detect a 20% increase in the notification of any recent partner(s).

### Statistical Analysis

For the preplanned primary analysis, we recategorized the study arms as follows: (1) Web-based PN (arms 1 and 2); (2) referral cards (arms 2 and 3); and (3) control (arm 4). This approach allowed us to maximize the use of limited resources by assessing two different PN tools within a single clinical trial design, although it was not powered to assess for a synergistic interaction between the interventions [40,41]. Descriptive characteristics for each study arm were calculated with medians and interquartile ranges (IQRs) for continuous variables and proportions for categorical variables. The proportion of participants who reported notifying any recent partner was calculated by the study arm. Next, ORs comparing “Web-based PN versus control” and “referral cards versus control” were calculated with a logistic regression model. We calculated the percentage of all partners, all male partners, stable male partners, and casual male partners who were notified by dividing the total number of partners reported per category by the number of partners per category that was notified. Due to the small number of female partners reported, female partners were excluded from the analysis. Then, we compared the percentage of partners notified in the “Web-based notification versus control” and “referral cards versus control” using the Wilcoxon rank-sum test. For analysis of data obtained from the participants’ 3 most recent partners, we used a logistic generalized estimating equation model to assess the notification and treatment outcomes for “Web-based notification versus control” and “referral cards versus control.” These outcomes specified (1) if the partner was notified, (2) if the participant knows that the partner received the message, (3) if the partner was known to have been tested for HIV and other STIs, and (4) if the partner was known to have received antibiotic treatment (either delivered by the participant or from another source). No interim analyses were conducted. All analyses were intention-to-treat, and all *P* values were two-sided. All statistical analyses were conducted in Stata 14.1 (StataCorp, College Station, TX, USA).

### Human Subjects Protections

All study procedures were reviewed and approved by the University of California, Los Angeles Office for Human Research Participant Protection (institutional review board #11-003105) and the Asociacion Civil Impacta Comite de Bioetica (Certificate #0052-2012-CE) and were registered with the *Peruvian Instituto Nacional de Salud* before the initiation of any activities. All participants underwent separate informed consent procedures for the screening and RCT protocols and provided written informed consent for each protocol. The clinical trial was registered with clinicaltrials.gov (Protocol Number NCT01720641).

## Results

**Figure 1:**
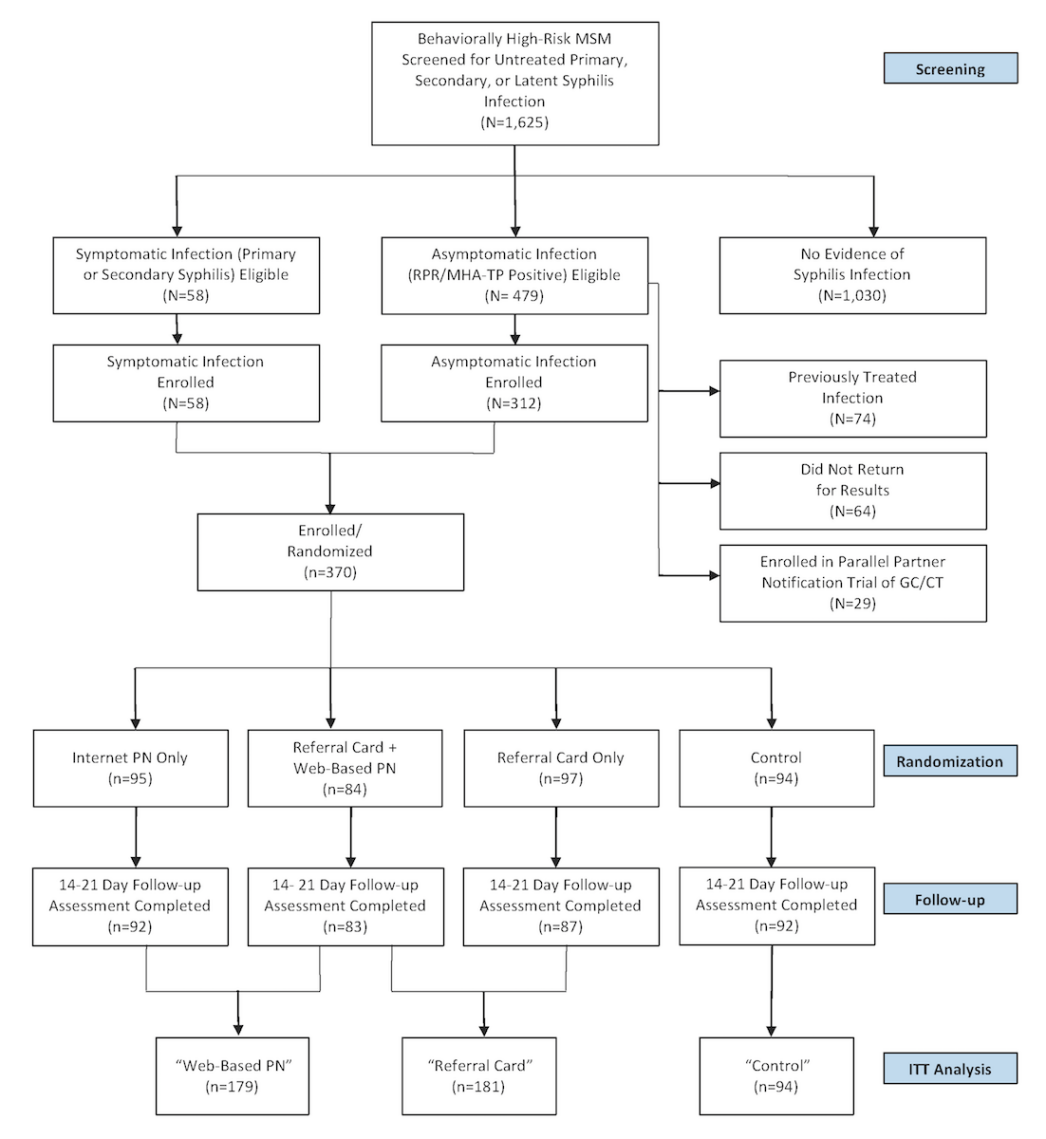
Screening, enrollment, and follow-up (CONSORT) flowchart; Lima, Peru 2012-2014. MSM: men who have sex with men; RPR/MHA-TP: rapid plasma reagin/microhemagglutination Treponema pallidum; GC/CT: gonorrhea/chlamydia; PN: partner notification; ITT: intention-to-treat.

Between November 2012 and June 2014, we screened 1625 individuals, of whom 537 were eligible for enrollment based on results of the physical examination or syphilis serology (Figure 1).

We noted signs and symptoms of primary syphilis in 36 individuals, whereas secondary infection was identified in 22 others. Of 479 MSM and TW with latent infection and positive RPR and MHA-TP assays, 74 were diagnosed with a previously treated infection that did not require additional treatment, 64 did not return for their results, and 29 were enrolled in a concomitant trial of expedited partner therapy for gonorrhea or chlamydia infection [42]. We enrolled 370 MSM or TW with recently diagnosed syphilis infection and randomly assigned each to one of the following four arms: (1) standard counseling or control (arm 1; N=94), (2) referral cards (arm 2; N=97), (3) Web-based PN (arm 3; N=95), or (4) combination Web-based PN and referral cards (arm 4; N=84). According to the prespecified analysis plan, data from arms 2 and 4, as well as arms 3 and 4, were combined into single arms (“referral cards” and “Web-based PN,” respectively).

The median age of participants was 30 years in the control group and 27 years in the other two arms (Table 1). The majority of participants had completed secondary school, as well as some university education or technical training, and reported daily or weekly internet use. The most commonly cited sexual identity was “gay or homosexual,” with most participants describing their role during intercourse as Moderno (versatile) or Pasivo (receptive). Participants reported a median of 2 male or transgender female sexual partners during the past 30 days. Symptomatic syphilis was more frequently noted among participants in the referral cards (38/181, 21.0%) than the Web-based PN (29/179, 16.2%) and control arms (14/94, 14.8%). Furthermore, the median RPR titer ranged from 1:16 in the Web-based PN arm to 1:32 in the other arms.

At 14-21 Days Follow-up, the proportion of subjects who reported notifying any recent sexual partners of their diagnosis was significantly lower in the Control arm (49/94; 52.1%) than in both the Referral Cards (117/181; 64.6%; Odds Ratio and 95% Confidence Interval: 1.68 [1.01-2.79]) and the Web-based PN (126/179; 70.4%; OR and 95% CI: 2.18 [1.30-3.36]) arms (Table 2).

**Table 1:** Baseline characteristics by randomization arms (N=370); Lima, Peru, 2012-2014. Arms 1 and 2: Web-Based partner notification (PN; N=179); Arms 2 and 3: Referral Cards (N=181); Arm 4: Control (n=94).

Demographic		Arm 1: Web-based PN only (n=95)	Arm 2: Referral cards + Web-based PN (n=84)	Arm 3: Referral cards only (n=97)	Arm 4: Control (counseling; n=94)
Age (years), median (IQR^a^)		27 (23-34)	27 (23-35)	26 (23-30)	30 (24-35)
**Education, n (%)**					
	Primary school only	2 (2.1%)	0 (0%)	0 (0%)	2 (2.2%)
	Incomplete secondary school	9 (9.5%)	9 (10.8%)	11 (11.3%)	11 (12.1%)
	Complete secondary school	20 (21.0%)	23 (27.4%)	25 (25.8%)	24 (25.5%)
	University or vocational training	64 (67.4%)	52 (61.9%)	61 (62.9%)	57 (60.1%)
**Sexual identity, n (%)**					
	Heterosexual	3 (3.2%)	0 (0%)	2 (2.1%)	3 (3.2%)
	Bisexual	13 (13.7%)	25 (29.8%)	22 (22.6%)	20 (21.3%)
	Homosexual/gay	71 (74.7%)	55 (65.4%)	67 (69.1%)	67 (71.3%)
	Trans	2 (2.1%)	1 (1.2%)	0 (0%)	2 (2.1%)
	Other	0 (0%)	1 (1.2%)	3 (3.1%)	2 (2.1%)
	I don’t know	6 (6.3%)	2 (2.4%)	4 (4.1%)	0 (0%)
**Sexual role, n (%)**					
	*Activo* (Insertive)	14 (14.7%)	13 (15.5%)	12 (12.4%)	12 (12.8%)
	*Pasivo* (Receptive)	28 (29.5%)	21 (25.0%)	35 (36.0%)	22 (23.4%)
	*Moderno* (Versatile)	47 (49.5%)	49 (58.3%)	43 (44.3%)	57 (60.6%)
	Other	4 (4.2%)	1 (1.2%)	3 (3.1%)	2 (2.1%)
	I don’t know	2 (6.3%)	0 (0%)	4 (4.1%)	1 (1.1%)
**Number of sexual partners (past 30 days), median (IQR)**		2 (1-3)	2 (1-3)	3 (1-5)	3 (1-5)
	Number of male partners	2 (1-3)	2 (1-4)	3 (1-5)	2 (1-4)
	Number of female partners	0 (0-0)	0 (0-0)	0 (0-0)	0 (0-0)
**Frequency of internet use, n (%)**					
	Daily	69 (72.6%)	64 (76.2%)	68 (70.1%)	69 (73.4%)
	Weekly	17 (17.9%)	17 (20.2%)	18 (18.6%)	17 (18.1%)
	Monthly	4 (4.2%)	0 (0%)	4 (4.1%)	3 (3.2%)
	Less than monthly	3 (3.2%)	1 (1.2%)	2 (2.1%)	2 (2.1%)
	Never	2 (2.1%)	2 (2.4%)	5 (5.2%)	3 (3.2%)
RPR^b^ titer, median (IQR)		16 (8-64)	16 (8-64)	32 (8-64)	32 (8-64)
**Symptomatic infection, n (%)**					
	Primary syphilis	10 (10.5%)	14 (16.7%)	14 (14.4%)	7 (7.4%)
	Secondary syphilis	3 (3.2%)	2 (2.4%)	8 (8.2%)	7 (7.4%)
	Latent syphilis	82 (86.3%)	68 (80.9%)	75 (77.4%)	80 (85.2%)

^a^IQR: interquartile range.

^b^RPR: rapid plasma reagin.

**Table 2:** Partner notification outcomes among men who have sex with men with recently diagnosed syphilis; Lima, Peru; 2012-2014.

	Percentage who notified any recent partners, n (%)	OR^a^ (95% CI)	Percentage who notified any recent partners (≥1 recent partner), n (%)	OR (95% CI)
Arm 1: Web-based PN^b^ only (n=95)	62/95 (65.2)		62/86 (72.1)	
Arm 2: Referral cards only (n=97)	53/97 (54.6)		53/79 (67.1)	
Arm 3: Referral cards and Web-based PN (n=84)	64/84 (76.2)		64/73 (87.7)	
Arm 4: Control (n=94)	49/94 (52.1)	—	49/79 (62.0)	—
Arms 1+3: All Web-based PN (N=179)	126/179 (70.4)	2.18 (1.30-3.66)	126/159 (79.2)	2.34 (1.29-4.24)
Arms 2+3: All referral cards (N=181)	117/181 (64.6)	1.68 (1.01-2.79)	117/152 (77.0)	2.05 (1.13-3.70)

^a^OR: odds ratio. Arm 4: Control is the reference category for all ORs.

^b^PN: partner notification.

After excluding individuals who denied having any sexual partners in the 30 days before enrollment, the strength of the correlation between the intervention arm and the PN outcomes increased further (referral cards OR 2.05, 95% CI 1.13-3.70; Web-based PN OR 2.34, 95% CI 1.29-4.24).

**Figure 2:**
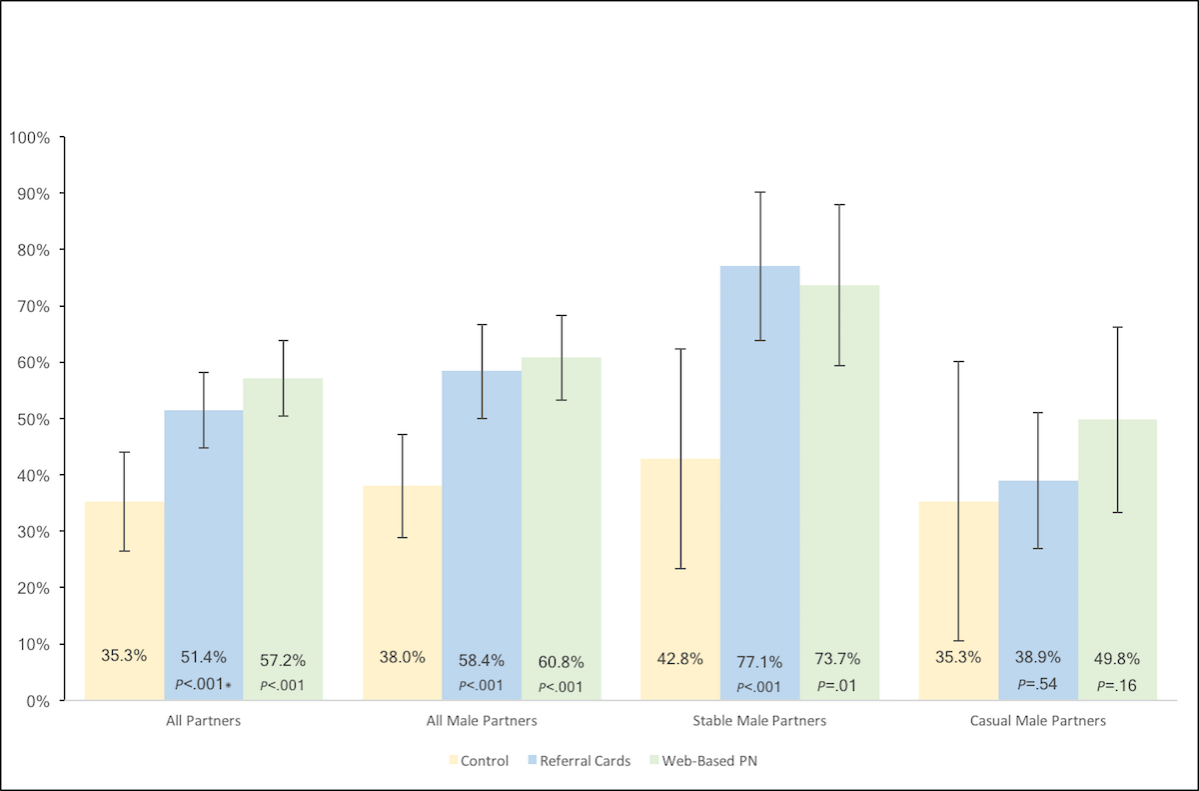
The proportion of all recent partners notified among men who have sex with men diagnosed with syphilis infection; Lima, Peru 2012-2014. PN: partner notification; *: Calculated using the Rank-Sum Test..

The proportion of participants’ 3 most recent partners who had been notified was significantly higher among those assigned to the referral cards (240/467, 51.4%; 95% CI 44.7%-58.1%; *P*<.001) and Web-based PN (241/421, 57.2%; 95% CI 50.5%-63.9%; *P*<.001) interventions than among those in the control arm (82/232, 35.3%; 95% CI 26.5%-44.1%; Figure 2). This observed difference in self-reported notification outcomes remained significant when limited to all male partners (*P*<.001) and stable male partners (*P*=.01). Although the frequency of notification for casual male partners was significantly higher in both intervention arms, these differences did not attain statistical significance (*P*=.16 and *P*=.54).

**Figure 3:**
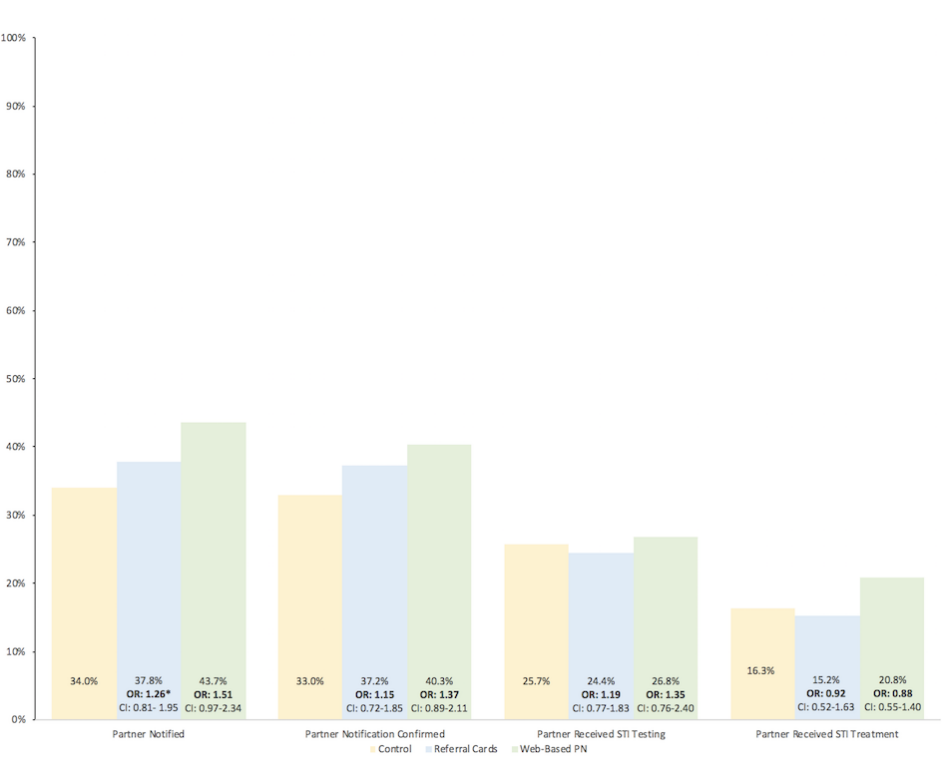
Prevention cascade outcomes of 3 most recent partners of MSM diagnosed with syphilis infection; Lima, Peru 2012-2014. PN: patient notification; STI: sexually transmitted infection. *Generalized Estimating Equation Model.

The availability of the intervention tools resulted in higher reported frequencies of attempted notification, confirmed notification, and partner STI testing for participants’ 3 most recent partners, although none of these comparisons was statistically significant (Figure 3). Furthermore, the frequency of participant-reported partner STI treatment was highest in the Web-based PN arm; however, the proportion of partners known to have received treatment was higher in the control arm than in the referral cards arm.

During the study period, the Peru section of the inSPOT website received 183 unique visits. These visitors sent a total of 47 different e-card messages to 119 recipients (allowing for the possibility of sending the same card to multiple recipients). No episodes of interpersonal violence or partner abuse were reported by study participants in any of the arms.

## Discussion

In this study, both new Web-based and traditional printed-media notification tools resulted in significant increases in self-reported PN outcomes compared with simple counseling. Peruvian MSM with newly diagnosed syphilis who were randomly assigned to receive printed referral cards and/or access to a Web-based notification system were more likely to report that they had notified at least one recent sexual partner and to report a larger proportion of recent sex partners. While the greatest impact on notification outcomes was observed among stable partners, smaller increases in notification were also observed with casual partners, particularly among participants randomly assigned to receive access to the Web-based PN system. These findings provide important data to guide global STI control efforts and suggest the potential importance of simple, inexpensive methods to promote patient-directed PN by MSM in resource-limited settings.

To the best of our knowledge, this study is the first to provide RCT evidence of the effectiveness of patient-controlled, Web-based notification systems among MSM. The odds of notifying a recent sexual partner of a potential syphilis exposure were significantly higher among men who received access to the PN website. These results are consistent with findings from our formative research and imply an absolute increase of 30% in the proportion of stable male partners notified and 15% among casual male partners [30]. A synthesis of this data with our formative research findings reveals that a positive effect of Web-based systems on notification outcomes could be attributed to multiple factors, including the availability of an anonymous mechanism to notify partners of a potentially stigmatizing diagnosis, the ability to contact partners for whom only an email address is known, and the opportunity to replicate existing methods of communication among young people already accustomed to communicating through Web-based networks [39,43].

Although extensive research has been conducted on the effect of printed referral cards for PN, this study is one of the first to evaluate their use within MSM partnerships in Latin America. As with Web-based systems, participants randomly assigned to receive printed partner referral cards exhibited a higher likelihood of notifying at least one partner and a considerably larger proportion of all their recent partners. The observed effect of referral card distribution was highest among stable male partners, with only a minimal effect on the number of casual male partners notified. These findings suggest that notification tools, such as partner referral cards, could be the most valuable in the context of established partnerships, where face-to-face communication is more likely, as opposed to casual sexual contacts where direct, in-person communication is often rare or nonexistent. Taken together, our data support the routine use of these simple, inexpensive tools as resources to enhance patient-driven notification in LMIC settings or other contexts where provider-guided notification programs are unavailable.

In contrast to the substantial impact of both intervention tools on the frequency of notification and the proportion of partners notified, data on more distal outcomes in the notification cascade, such as partner testing and STI treatment, were inconclusive. The assessment of outcomes limited to participants’ 3 most recent sexual partners revealed a nonsignificant increase in the participant-reported notification and confirmation of notification, but minor, or even negative, differences in the frequency of partner STI testing and treatment (as reported by participants). As this study was designed to promote and assess only the initial step of notification, the small number of partners included in this section of the analysis and the use of generalized estimating equation modeling to control for multiple observations per participant is likely to have limited our power to detect statistically significant differences. In addition, the dissipation of the intervention effect observed as we progressed through the partner management cascade might reflect the participants’ inability to follow up on their notification messages, particularly the ones sent to casual partners through an anonymous, Web-based system. The lack of an observed effect on downstream outcomes indicates the need for additional interventions to support partner management outcomes throughout the partner management cascade, beginning with notification and culminating with a linkage to and retention in HIV or STI care [42]. While the preliminary research on potential responses to hypothetical anonymous notification messages among MSM in Peru indicated that the recipients would be motivated to seek medical attention after receiving an anonymous message, data from the United States have suggested otherwise, and this study too did not collect any partner-confirmed information on postnotification behavior [33,43]. Additional research is needed to evaluate these hypotheses and support both index cases and their partners throughout the HIV or STI prevention and treatment process.

There are several limitations to be considered when interpreting our findings. First, the fact that our data are based on participants’ self-report, without independent confirmation by sexual partners, increases the possibility that observed improvements in notification might have been due to social desirability bias in reporting. However, this bias would have likely affected all arms of the study, resulting in a type II error in favor of the null hypothesis and thereby underestimated the true impact of these notification tools. In order to minimize the possibility of desirability bias, we enrolled participants using standardized scripts informing them only that they were invited to a study of, “If and how men with an STI inform their recent sexual partners of their diagnosis,” without specifying the different intervention tools being evaluated. In addition, all participants, regardless of the intervention arm, were read the same standardized counseling script advising on the importance of notification and the availability of local testing and treatment resources. Due to the lack of independent partner confirmation, data on the more distal outcomes in the prevention cascade (eg, partner testing and antibiotic treatment) are less reliable and require further investigation with directly confirmed outcomes to be validated. As we did not survey partners on notification outcomes, we were unable assess the cross-contamination between study arms and determine whether individual partners received notification from multiple sources. Similarly, given the relatively small sample size of this study, secondary evaluations of participant- and partner-level factors that might have modified the effect of PN technologies, including the presence or absence of biological symptoms, new diagnosis of HIV coinfection, and differences in the gender and sexual identities of participants and their partners, are beyond the scope of the data presented. Finally, as few TW were enrolled in this study and few cisgender female partners were reported, we did not have sufficient data to draw any conclusions regarding notification by or to MSM and these other groups. Despite these limitations, this study provides important preliminary data to support research into new methods for PN following STI diagnosis in resource-limited settings.

This study provides critical clinical trial evidence to support the effectiveness of both new and traditional notification technologies to support patient-directed PN among MSM with syphilis in Latin America. Regarding the global resurgence in the syphilis incidence within MSM sexual networks, accompanied by endemic levels of transmission among MSM in Latin America, these tools offer simple, inexpensive resources that can dramatically affect the frequency of PN following syphilis diagnosis. While provider-initiated notification by dedicated health professionals is highly effective in promoting PN, testing, and treatment, several resource-limited health systems lack the workforce to implement provider-based notification systems. As a result, strategies to support patient-initiated notification are critical to controlling the disseminated syphilis epidemics that exist among MSM and their male, female, and transgender partners in Latin America. Additional research is needed to explore partner responses to patient-initiated notification messages and develop effective interventions to support testing and treatment outcomes throughout the partner management cascade. Both Web-based notification systems and printed partner referral cards offer simple, effective tools to support the first step in PN, testing, and linkage to care cascade and can fill a unique and essential niche for global HIV and STI prevention efforts.

## Abbreviations

CASI: computer-based self-interviewing
GC/CT: gonorrhea/chlamydia
IQR: interquartile range
ITT: intention-to-treat
LMIC: low- and middle-income countries
MHA-TP: microhemagglutination Treponema pallidum
MSM: men who have sex with men
PN: partner notification
RCT: randomized controlled trials
RPR: rapid plasma reagin
STI: sexually transmitted infection
TW: transgender women

## Appendix

Multimedia Appendix 1 CONSORT-EHEALTH checklist (V 1.6.1).

## References

[1] Parran Thomas (1937). Shadow on the Land: Syphilis.

[2] Hawkes S, Mabey D, Mayaud P (2003). Partner notification for the control of sexually transmitted infections. BMJ.

[3] Hogben M (2007). Partner notification for sexually transmitted diseases. Clin Infect Dis.

[4] Clark J, Salvatierra J, Segura E, Salazar X, Konda K, Perez-Brumer A, Hall E, Klausner J, Caceres C, Coates T (2013). Moderno love: sexual role-based identities and HIV/STI prevention among men who have sex with men in Lima, Peru. AIDS Behav.

[5] Rothenberg R (2002). The transformation of partner notification. Clin Infect Dis.

[6] Wohlfeiler D, Potterat JJ (2005). Using gay men's sexual networks to reduce sexually transmitted disease (STD)/human immunodeficiency virus (HIV) transmission. Sex Transm Dis.

[7] Hogben M, Paffel J, Broussard D, Wolf W, Kenney K, Rubin S, George D, Samoff E (2005). Syphilis partner notification with men who have sex with men: a review and commentary. Sex Transm Dis.

[8] Tan WS, Chen M, Ivan M, Stone K, Rane V, Fairley CK, Ong JJ (2016). Partner Notification Outcomes for Men Who Have Sex With Men Diagnosed With Syphilis Referred to Partner Notification Officers, Melbourne, Australia. Sex Transm Dis.

[9] de Lorenzi C, Gayet-Ageron A, Girard-Strohbach M, Toutous-Trellu L (2017). Tracing partners of patients with syphilis infection remains challenging: experience of Geneva Hospital. Int J STD AIDS.

[10] Ferreira A, Young T, Mathews C, Zunza M, Low N (2013). Strategies for partner notification for sexually transmitted infections, including HIV. Cochrane Database Syst Rev.

[11] Alam N, Chamot E, Vermund SH, Streatfield K, Kristensen S (2010). Partner notification for sexually transmitted infections in developing countries: a systematic review. BMC Public Health.

[12] Clark JL, Long CM, Giron JM, Cuadros JA, Caceres CF, Coates TJ, Klausner JD, NIMH Collaborative HIV/STD Prevention Trial (2007). Partner notification for sexually transmitted diseases in Peru: knowledge, attitudes, and practices in a high-risk community. Sex Transm Dis.

[13] Wang AL, Peng R, Tucker JD, Cohen MS, Chen X (2012). Partner notification uptake for sexually transmitted infections in China: a systematic literature review. Sex Transm Infect.

[14] Semple SJ, Pines HA, Strathdee SA, Vera AH, Rangel G, Magis-Rodriguez C, Patterson TL (2017). Uptake of a Partner Notification Model for HIV Among Men Who Have Sex With Men and Transgender Women in Tijuana, Mexico. AIDS Behav.

[15] Bilardi JE, Fairley CK, Hopkins CA, Hocking JS, Temple-Smith MJ, Bowden FJ, Russell DB, Pitts M, Tomnay JE, Parker RM, Pavlin NL, Chen MY (2010). Experiences and outcomes of partner notification among men and women recently diagnosed with Chlamydia and their views on innovative resources aimed at improving notification rates. Sex Transm Dis.

[16] Rietmeijer C (2013). Using social media for partners services in adolescents. Sex Transm Infect.

[17] Shiely F, Hayes K, Thomas KK, Kerani RP, Hughes JP, Whittington WLH, Holmes KK, Handsfield HH, Hogben M, Golden MR (2010). Expedited partner therapy: a robust intervention. Sex Transm Dis.

[18] Trelle S, Shang A, Nartey L, Cassell JA, Low N (2007). Improved effectiveness of partner notification for patients with sexually transmitted infections: systematic review. BMJ.

[19] Kissinger P, Schmidt N, Mohammed H, Leichliter JS, Gift TL, Meadors B, Sanders C, Farley TA (2006). Patient-delivered partner treatment for Trichomonas vaginalis infection: a randomized controlled trial. Sex Transm Dis.

[20] Schillinger JA, Kissinger P, Calvet H, Whittington WLH, Ransom RL, Sternberg MR, Berman SM, Kent CK, Martin DH, Oh MK, Handsfield HH, Bolan G, Markowitz LE, Fortenberry JD (2003). Patient-delivered partner treatment with azithromycin to prevent repeated Chlamydia trachomatis infection among women: a randomized, controlled trial. Sex Transm Dis.

[21] Díaz OC, Valencia J, Wilson K, García SG, Tinajeros F, Sanchez T (2011). Assessing the effectiveness of a patient-driven partner notification strategy among pregnant women infected with syphilis in Bolivia. Sex Transm Infect.

[22] Hogben M, Collins D, Hoots B, OʼConnor K (2016). Partner Services in Sexually Transmitted Disease Prevention Programs: A Review. Sex Transm Dis.

[23] Low N, McCarthy A, Roberts TE, Huengsberg M, Sanford E, Sterne JAC, Macleod J, Salisbury C, Pye K, Holloway A, Morcom A, Patel R, Robinson SM, Horner P, Barton PM, Egger M (2006). Partner notification of chlamydia infection in primary care: randomised controlled trial and analysis of resource use. BMJ.

[24] Hopkins CA, Temple-Smith MJ, Fairley CK, Pavlin NL, Tomnay JE, Parker RM, Bowden FJ, Russell DB, Hocking JS, Chen MY (2010). Telling partners about chlamydia: how acceptable are the new technologies?. BMC Infect Dis.

[25] Mimiaga MJ, Fair AD, Tetu AM, Novak DS, Vanderwarker R, Bertrand T, Adelson S, Mayer KH (2008). Acceptability of an internet-based partner notification system for sexually transmitted infection exposure among men who have sex with men. Am J Public Health.

[26] Tomnay JE, Gebert RL, Fairley CK (2006). A survey of partner notification practices among general practitioners and their use of an internet resource for partner notification for Chlamydia trachomatis. Sex Health.

[27] Bilardi JE, Fairley CK, Hopkins CA, Hocking JS, Sze JK, Chen MY (2010). Let Them Know: evaluation of an online partner notification service for chlamydia that offers E-mail and SMS messaging. Sex Transm Dis.

[28] Götz HM, van RMS, Vriens P, Op DCE, Hamers M, Heijman T, van DHF, Koekenbier R, van LAP, Voeten HACM (2014). Initial evaluation of use of an online partner notification tool for STI, called 'suggest a test': a cross sectional pilot study. Sex Transm Infect.

[29] van RM, Vriens P, Gotz H, Heijman T, Voeten H, Koekenbier R (2013). Acceptance of an onling partner notification tool for STI. called Suggest-a-Test. Sex Transm Infect.

[30] Clark JL, Segura ER, Perez-Brumer AG, Reisner SL, Peinado J, Salvatierra HJ, Sanchez J, Lama JR (2014). Potential impact and acceptability of Internet partner notification for men who have sex with men and transgender women recently diagnosed as having sexually transmitted disease in Lima, Peru. Sex Transm Dis.

[31] Plant A, Rotblatt H, Montoya JA, Rudy ET, Kerndt PR (2012). Evaluation of inSPOTLA.org: an Internet partner notification service. Sex Transm Dis.

[32] Rietmeijer CA, Westergaard B, Mickiewicz TA, Richardson D, Ling S, Sapp T, Jordan R, Wilmoth R, Kachur R, McFarlane M (2011). Evaluation of an online partner notification program. Sex Transm Dis.

[33] Kerani RP, Fleming M, Golden MR (2013). Acceptability and intention to seek medical care after hypothetical receipt of patient-delivered partner therapy or electronic partner notification postcards among men who have sex with men: the partner's perspective. Sex Transm Dis.

[34] Hottes TS, Gilbert M (2012). Evaluation of online partner notification services like inSPOT requires starting with the client, not the clinic. Sex Transm Dis.

[35] García PJ, Williams E, Cárcamo CP, Chiappe M, Holmes KK, Peeling RW, Mabey DM (2015). Partner Notification Among Peruvian Pregnant Women With Syphilis. Sex Transm Dis.

[36] Ministerio de Salud del Peru (2004). Guias Nacionales de Atencion Integral de Salud Sexual y Reproductiva. Guias Nacionales de Atencion Integral de Salud Sexual y Reproductiva.

[37] Braun HM, Segura ER, Lake JE, Gandhi M, Rios J, Villaran MV, Sanchez J, Lama JR, Clark JL (2017). Individual and partnership factors associated with anticipated versus actual partner notification following STI diagnosis among men who have sex with men and/or with transgender women in Lima, Peru. Sex Transm Infect.

[38] Long CM, Klausner JD, Leon S, Jones FR, Giron M, Cuadros J, Pajuelo J, Caceres C, Coates TJ, NIMH Collaborative HIV/STD Prevention Trial Group (2006). Syphilis treatment and HIV infection in a population-based study of persons at high risk for sexually transmitted disease/HIV infection in Lima, Peru. Sex Transm Dis.

[39] Clark JL, Perez-Brumer AG, Segura ER, Salvatierra HJ, Sanchez J, Lama JR (2016). Anticipated Notification of Sexual Partners following STD Diagnosis among Men Who Have Sex with Men and Transgender Women in Lima, Peru: A Mixed Methods Analysis. PLoS One.

[40] McAlister FA, Straus SE, Sackett DL, Altman DG (2003). Analysis and reporting of factorial trials: a systematic review. JAMA.

[41] Pocock SJ, Clayton TC, Stone GW (2015). Challenging Issues in Clinical Trial Design: Part 4 of a 4-Part Series on Statistics for Clinical Trials. J Am Coll Cardiol.

[42] Clark JL, Segura ER, Oldenburg CE, Rios J, Montano SM, Perez-Brumer A, Villaran M, Sanchez J, Coates TJ, Lama JR (2017). Expedited Partner Therapy (EPT) increases the frequency of partner notification among MSM in Lima, Peru: a pilot randomized controlled trial. BMC Med.

[43] Clark JL, Perez-Brumer A, Salazar X (2015). “Manejar la Situacion”: Partner Notification, Partner Management, and Conceptual Frameworks for HIV/STI Control Among MSM in Peru. AIDS Behav.

